# Nanoliposomal VEGF-R2 peptide vaccine acts as an effective therapeutic vaccine in a murine B16F10 model of melanoma

**DOI:** 10.1186/s12645-023-00213-7

**Published:** 2023-06-14

**Authors:** Fatemeh Zahedipour, Parvin Zamani, Mohammad Mashreghi, Mojgan Astaneh, Mojtaba Sankian, Atefeh Amiri, Khadijeh Jamialahmadi, Mahmoud Reza Jaafari

**Affiliations:** 1 Department of Medical Biotechnology and Nanotechnology, Faculty of Medicine, Mashhad University of Medical Sciences, Mashhad, Iran; 2 Nanotechnology Research Center, Pharmaceutical Technology Institute, Mashhad University of Medical Sciences, Mashhad, Iran; 3 Immunology Research Center, School of Medicine, Mashhad University of Medical Sciences, Mashhad, Iran; 4 Biotechnology Research Center, Pharmaceutical Technology Institute, Mashhad University of Medical Sciences, Mashhad, Iran; 5 Department of Pharmaceutical Nanotechnology, School of Pharmacy, Mashhad University of Medical Sciences, Mashhad, Iran

**Keywords:** Nanoliposomal vaccine, VEGFR-2, Peptide vaccine, Melanoma

## Abstract

**Background:**

The vascular endothelial growth factor receptor-2 (VEGFR-2) plays an important role in melanoma development and progression. Peptide vaccines have shown great potential in cancer immunotherapy by targeting VEGFR-2 as a tumor-associated antigen and boosting the immune response against both tumor cells and tumor endothelial cells. Despite this, the low efficiency of peptide vaccines has resulted in moderate therapeutic results in the majority of studies. Enhancing the delivery of peptide vaccines using nanoliposomes is an important strategy for improving the efficacy of peptide vaccines. In this regard, we designed VEGFR-2-derived peptides restricted to both mouse MHC I and human HLA-A*02:01 using immunoinformatic tools and selected three peptides representing the highest binding affinities. The peptides were encapsulated in nanoliposomal formulations using the film method plus bath sonication and characterized for their colloidal properties.

**Results:**

The mean diameter of peptide-encapsulated liposomes was around 135 nm, zeta potential of − 17 mV, and encapsulation efficiency of approximately 70%. Then, vaccine formulations were injected subcutaneously in mice bearing B16F10-established melanoma tumors and their efficiency in triggering immunological, and anti-tumor responses was evaluated. Our results represented that one of our designed VEGFR-2 peptide nanoliposomal formulations (Lip-V1) substantially activated CD4^+^ (*p* < 0.0001) and CD8^+^ (*P* < 0.001) T cell responses and significantly boosted the production of IFN-γ (*P* < 0.0001) and IL-4 (*P* < 0.0001). Furthermore, this formulation led to a significant decrease in tumor volume (*P* < 0.0001) and enhanced survival (*P* < 0.05) in mice.

**Conclusion:**

Our findings suggest that the nanoliposomal formulation containing VEGFR-2 peptides could be a promising therapeutic vaccination approach capable of eliciting strong antigen-specific immunologic and anti-tumor responses.

**Supplementary Information:**

The online version contains supplementary material available at 10.1186/s12645-023-00213-7.

## Background

Melanoma is a cancer arising from melanocytes, which produce melanin (Rastrelli et al. 2014). Although being a rare form of skin cancer, it accounts for the great majority of skin cancer mortalities (Garbe et al. 2022). Moreover, metastatic melanoma is one of the most heterogeneous and aggressive cancers (Watson et al. 2015). Currently, surgery, chemotherapy, immunotherapy, and targeted therapy are the primary therapeutic options available to melanoma patients (Domingues et al. 2018). Depending on the stage of the disease, the location and genetic nature of the tumor, the patient’s overall health, and age, these treatments may be used as monotherapy or combination therapy. The development of immunotherapies has only recently led to a significant improvement in the progression-free and overall survival of melanoma patients (Kozar et al. 2019). Active immunotherapy techniques, such as immunization with epitope peptides derived from tumor-associated antigens (TAAs), have been successful in stimulating the immune response (Vergati et al. 2010; Lam et al. 2015). Peptide vaccines used in cancer immunotherapy have the ability to inhibit and reduce the growth of tumor cells within the host. Due to the ease and low cost of peptide synthesis and purification, peptide vaccination can be an appealing and straightforward method for stimulating the immune system. Additionally, these vaccination strategies are now available in research and clinical settings (He et al. 2018). TAAs that are produced by tumor cells are not easily recognized by the innate or acquired immune systems of humans because they are not very immunogenic. To enhance the recognition of tumor cells by immune cells and to decrease the activity of immunosuppressive cells like regulatory T cells (Tregs), it seems like activating the immune system effectively could be a viable strategy. (Kumai et al. 2017; Tsang et al. 2015). Successful activation of CD8^+^ cytotoxic T lymphocytes (CTLs) can lead to the effective elimination of tumors since cellular immunity is crucial for the removal of solid tumors (Tardón et al. 2019). The antigen must be delivered to CD8^+^ CTLs via MHC class I for efficient and potent activation of CTL response resulting in tumor suppression (Parkin and Cohen 2001).

Angiogenesis is a crucial process in many malignancies, including melanoma. The expression of vascular endothelial growth factor (VEGF) and VEGF receptors (VEGFRs) is essential for the development of the immunosuppressive tumor microenvironment (Roskoski Jr 2007). In particular, freshly produced tumor blood vessels display high levels of VEGFR-2, a functional protein linked to neovascularization, while normal vessels do not. Melanoma cells can gain the ability to overexpress the VEGFR-2 during the vascular mimicry phase, which is generally expressed in endothelial cells (Mahabeleshwar and Byzova 2007). Therefore, VEGFR-2-derived peptide vaccines are potent options for the treatment of melanoma since they target both tumor cells and endothelial cells while producing substantial anti-tumor immune responses with low toxicity (Mahabeleshwar and Byzova 2007; Zahedipour et al. 2021). Although specific immunity against VEGFRs can be enhanced in individuals inoculated with these peptides, clinical investigations have shown a significant result with relatively minimal side effects (Masuzawa et al. 2012; Yoshimura et al. 2013). One potential strategy to increase the efficacy of these peptide vaccines is designing a potent delivery system.

Liposomes have received a lot of interest recently as antigen and adjuvant carriers for vaccine development (Nikoofal-Sahlabadi et al. 2018; Zamani et al. 2018; Behravan et al. 2022; Gao et al. 2017). Liposomes are spherical vesicles consisting of various natural or synthesized phospholipids as well as different cholesterol ratios. Numerous properties of liposomes offer them desirable platforms for the development of vaccines. These properties include biocompatibility, biodegradability, low toxicity, amphipathic features, and immunogenicity (Akbarzadeh et al. 2013). Furthermore, liposomes’ size, chemical composition, and surface charge can be easily altered to efficiently target antigen-presenting cells (APCs) (Petrovic et al. 2021). Several studies have shown that liposomes are of considerable importance in the maturation of APCs and their antigen presentation capability and that they may enable enhance antigen cross-presentation in dendritic cells (DCs) to activate CD8^+^ T Cells, which have an essential role in triggering the immune response against tumors (Yazdani et al. 2021; Yuba 2020). It was also established that pH-sensitive liposomes, particularly ones constituted of Dioleoylphosphatidylethanolamine (DOPE), may release their content into the cytosol of DCs, eliciting specific cellular immunity against the antigen and cross-presentation via the MHC class I pathway (Yuba 2020). In this respect, liposomes can be regarded as an efficient delivery method of peptide antigens to stimulate cell-mediated antitumor immunity (Gu et al. 2020).

**Scheme 1:**
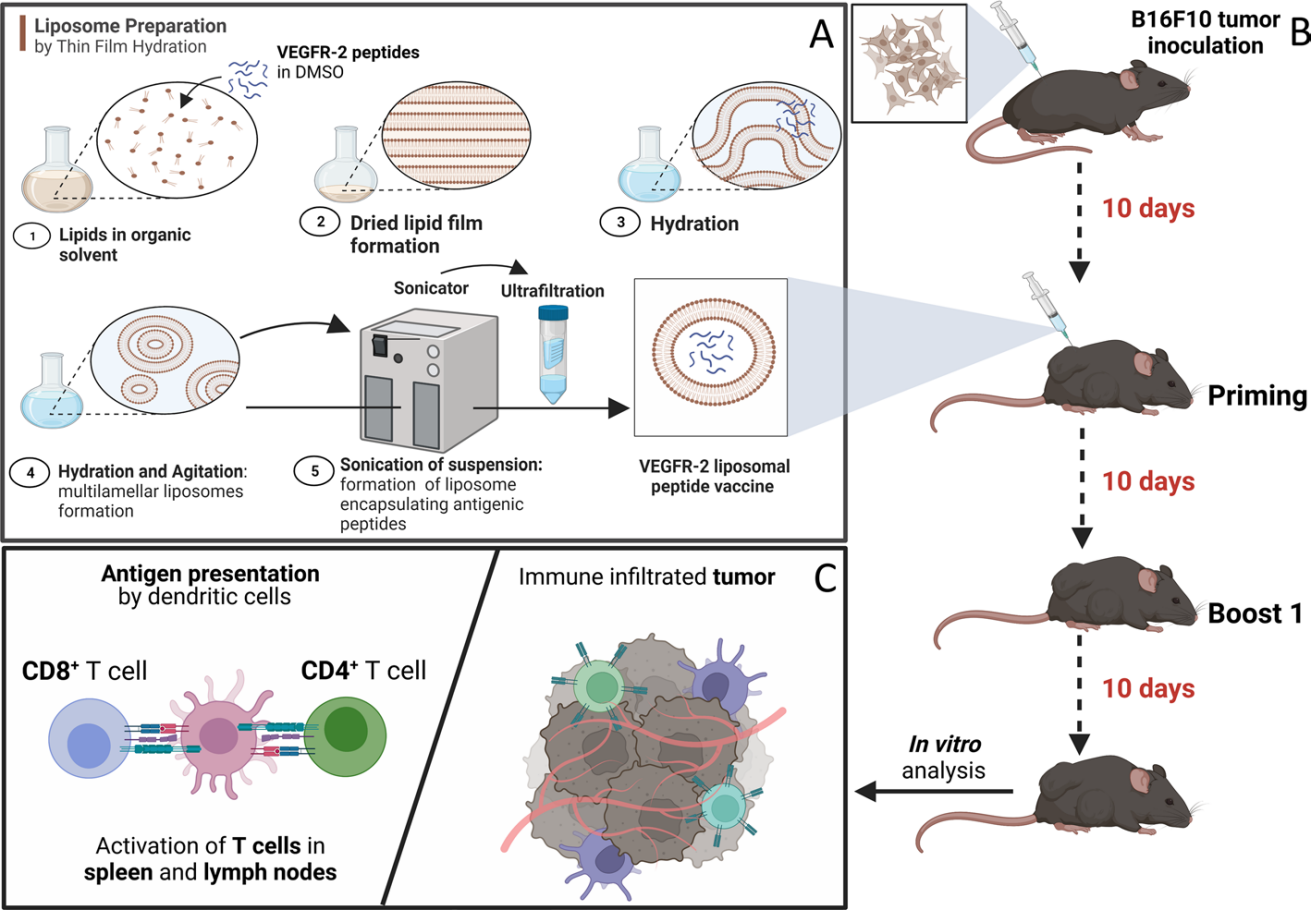
Schematic overview of the preparation of nanoliposomal peptide vaccine formulations using the film method plus bath sonication (**A**), animal immunization schedule (**B**), and resulted immune and anti-tumor responses by activated CD4 ^+^ and CD8^ +^ cells (**C**)

In this study, we first designed and selected VEGFR-2 epitope peptides by in silico analysis from the VEGFR-2 antigen (homologous to mouse and human VEGFR-2), which are capable of activating T-cells clones. VEGFR-2-derived peptides are poorly immunogenic and tolerated by the immune system because they are fragments of endogenous protein that are expressed by various cells. Therefore, we hypothesized that the encapsulation of VEGFR-2 peptides in nanoliposomal formulations might be a potential immunization strategy for enhancing the antitumor immunity against VEGFR-2 overexpressing melanoma tumors in the C57BL/6 mice model. In this regard, the peptides were encapsulated in nanoliposomes using the film method plus bath sonication, characterized for their colloidal properties, and evaluated for their potency in inducing immune and antitumor responses (Scheme 1). The information gathered by this study may be useful in the implementation of nanoliposomal peptide-based vaccines in clinical settings.

## Results

### Design of CTL peptide epitopes

According to the data obtained from the NetCTLpan, The Immune Epitope Database (IEDB), NetMHC 4.0, and PickPocket 1.1 servers designed peptides were ranked. Finally, three peptide sequences with nine amino acids length that simultaneously have the highest binding affinity to mouse MHC I (H-2-Db, H-2-Kb) and human HLA-A*02 were selected and named as V1, V2, and V3 peptides. The rank of each of peptides and their sequences are shown in Table 1.

**Table 1:** Selected CTL peptide epitopes and their scores

Database	Allele		Sequence	Start–End	Length (aa)^c^	MHC prediction		TAP prediction score		Combined prediction score		% Rank		Affinity (1-log50k)	
	HLA^a^	MHC^b^				HLA	MHC	HLA	MHC	HLA	MHC	HLA	MHC	HLA	MHC
NetCTLpan	HLA-A*02:01	H-2-Db H-2-kb	YMISYAGMV (V1)	189–198	9	0.861		0.539		0.95325		0.4			
	HLA-A*02:01	H-2-Db H-2-kb	YTVILTNPI (V2)	396–404	9	0.394		0.712		0.6235		3			
	HLA-A*02:01	H-2-Db H-2-kb	IQAANVSAL (V3)	519–527	9	0.441		1.117		0.58019		4			
IEDB	HLA-A*02:01	H-2-Db H-2-kb	YMISYAGMV (V1)	189–198	9	− 2.16	− 1.98	0.24	0.23	− 0.96	− 0.86				
	HLA-A*02:01	H-2-Db H-2-kb	YTVILTNPI (V2)	396–404	9	− 2.65	− 2.48	0.31	0.31	− 1.57	− 1.26				
	HLA-A*02:01	H-2-Db /H-2-kb	IQAANVSAL (V3)	519–527	9	− 2.6	− 2.04	0.49	0.49	− 0.85	− 0.29				
NetMHC 4.0	HLA-A*02:01	H-2-Db H-2-kb	YMISYAGMV (V1)	189–198	9			`				0.5	2	0.828	0.483
	HLA-A*02:01	H-2-Db H-2-kb	YTVILTNPI (V2)	396–404	9							3	0.1	0.435	0.515
	HLA-A*02:01	H-2-Db H-2-kb	IQAANVSAL (V3)	519–527	9							3.5	0.12	0.4	0.499
PickPocket 1.1	HLA-A*02:01	H-2-Db H-2-kb	YMISYAGMV (V1)	189–198	9									0.796	0.413
	HLA-A*02:01	H-2-Db H-2-kb	YTVILTNPI (V2)	396–404	9									0.434	0.407
	HLA-A*02:01	H-2-Db H-2-kb	IQAANVSAL (V3)	519–527	9									0.492	0.466

^a^Human leukocyte antigen

^b^Major histocompatibility complex

^C^Amino acid

### Evaluation of peptides

The evaluation of some features of the peptides including their molecular weight, pI value, stability, aliphatic, and hydrophobic indexes was performed by the ProtParam server. According to the data obtained from the server, the molecular weight of the V1, V2, and V3 peptide vaccine candidates were 1034.25, 1033.23, and 886.02 Da, respectively; the predicted pI was 5.52 for all three peptides; and the evaluated half-life was shown that the peptides are stable. The evaluated aliphatic index of V1, V2, and V3 peptides were 82.67, 162.22, and 152.22, respectively which indicates that the vaccine is thermostable. GRAVY of V1, V2, and V3 peptides was calculated as 1.167, 1.022, and 1.122, respectively demonstrating the hydrophobic nature of peptides.

### Characterization of the nanoliposomes containing VEGFR-2 peptides

**Fig. 1:**
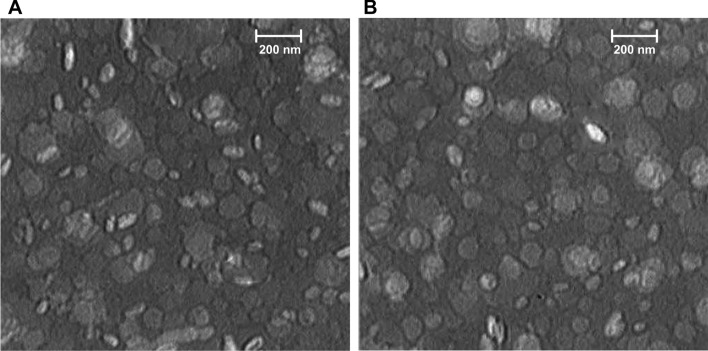
Transmission Electron Microscopy (TEM) images of liposomes staining with 2% uranyl acetate. **A** Naïve liposome formulation, **B** Nanoliposomal-peptide formulation (Lip-V1)

Characteristics of liposomal formulations, including size, PDI, zeta potential, and encapsulation efficiencies (EE%), are shown in Table 2. All nanoliposomes exhibited particle sizes between 128 and 168 nm with PDI < 0.3 and surface charge at about − 17 mV. Liposomal formulations showed spherical-shaped particles according to TEM images (Fig. 1). Moreover, the encapsulation efficiency (%EE) of peptides in liposomal formulations was approximately 70% (Additional file 1: Fig. S1).

**Table 2:** Physicochemical characteristics of the liposomal formulations

Formulation	Lipid composition	Lipid Molar Ratio	Z average (nm)^a^	Z potential (mV)^b^	PDI^c^	% EE^d^
Naïve Liposome	DMPC:DMPG:DOPE:Chol	60:8:20:12	128 ± 0.6	− 17 ± 0.1	0.28	–
Lip-V1	DMPC:DMPG:DOPE:Chol	60:8:20:12	138 ± 0.8	− 17 ± 0.1	0.26	74.1 ± 2.2
Lip-V2	DMPC:DMPG:DOPE:Chol	60:8:20:12	141 ± 0.9	− 29 ± 0.2	0.27	66.8 ± 4.7
Lip-V3	DMPC:DMPG:DOPE:Chol	60:8:20:12	139 ± 0.5	− 17 ± 0.4	0.27	74.8 ± 0.7

The results of triple measurements of each formulation are reported. The information is displayed as mean ± Standard deviation (S.D)

^a^The size of liposomes (Z average)

^b^The charge of liposomes

^c^Polydispersity index

^d^Encapsulation Efficiency (%)

### Tracking of nanoliposome migration into lymph nodes

**Fig. 2:**
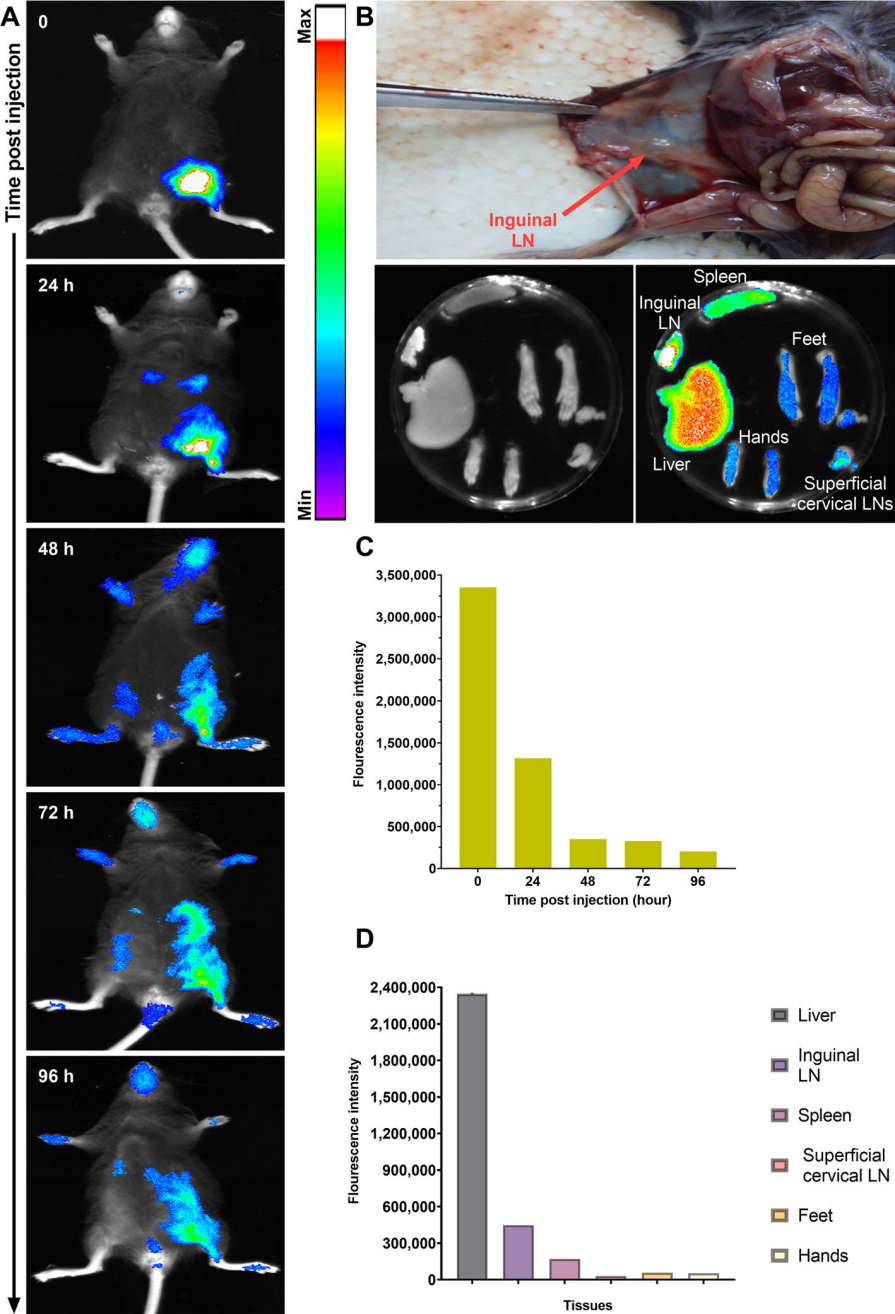
In vivo biodistribution of DiR-labeled nanoliposomes administered subcutaneously to mice. **A** Whole-body images of DiR-labeled liposomes at various time points after treatment. **B** Ex vivo imaging of mice liver, LNs, spleen, feet, and hands 96 h following the liposome injection. The quantitative fluorescence intensity of DiR-labeled liposomes throughout the body **C**, as well as the signal emitted by LNs and other organs **D**, was measured. The fluorescence intensity scale is also shown

The DiR-labeled liposomes were injected subcutaneously into the groin area, and DiR fluorescence was monitored over time. Immediately after injection, a strong fluorescent signal in the injection site could be detected followed by a significant decrease at 48 h. The formulation showed gradual dispersion in the body from 24 to 96 h (Fig. 2A, C). In addition to the injection site, strong fluorescent signals were detected in the inguinal lymph node (LN) at the injection site at 24 h (Fig. 2A). Once 48 h had elapsed, the fluorescence at the injection site began to diminish while the fluorescence in the other lymph nodes increased in response. When reaching 96 h, the mice were sacrificed and the liver, LNs (inguinal and superficial cervical), spleen, hands, and feet were collected. The highest fluorescence intensities were observed in the liver, spleen, and inguinal LN, respectively (Fig. 2B, D). The collected data provided evidence that the liposomal formulation was moving away from the injection site and toward the lymphatic sites.

### In vitro cellular uptake assay

**Fig. 3:**
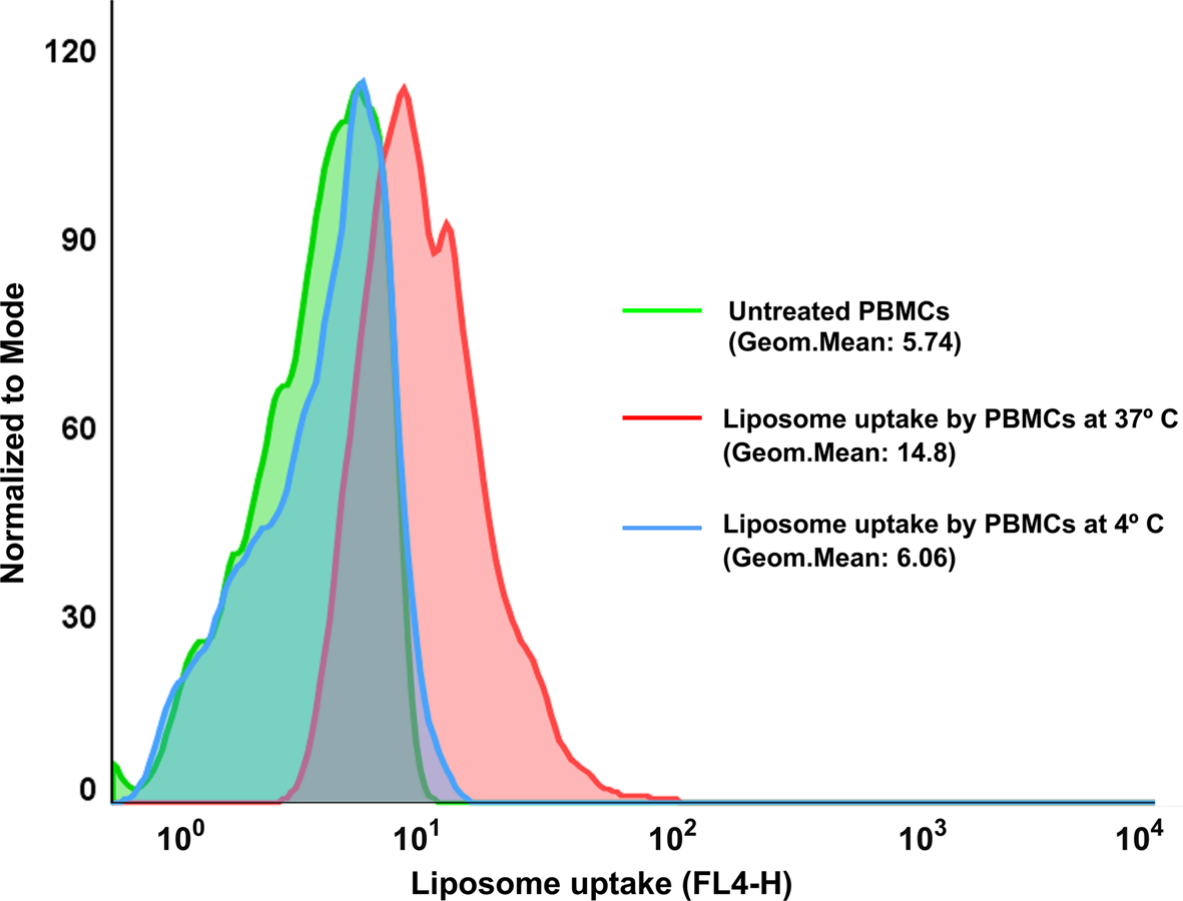
In vitro cellular uptake of DiR-labeled liposomes by PBMCs. PBMCs incubated with nanoparticles for 2 h either in 4 °C and 37 °C. The cellular uptake in PBMCs incubating with nanoliposomes at 4 °C was similar to that of untreated group, while PBMCs that were incubated at 37 °C showed a significant level of uptake

Mouse PBMC's nanoliposomal uptake was analyzed in vitro. The results indicated that at 37 °C, PBMCs could successfully uptake the liposomes. The uptake of PBMCs for naïve nanoliposomes at 37 °C was higher than that seen at 4 °C, as shown in Fig. 3, with mean fluorescence intensities of 14.8 and 6.06, respectively. These findings suggested that PBMCs can efficiently uptake liposomes in culture media.

### Analysis of the frequency of T cell sub-populations

**Fig. 4:**
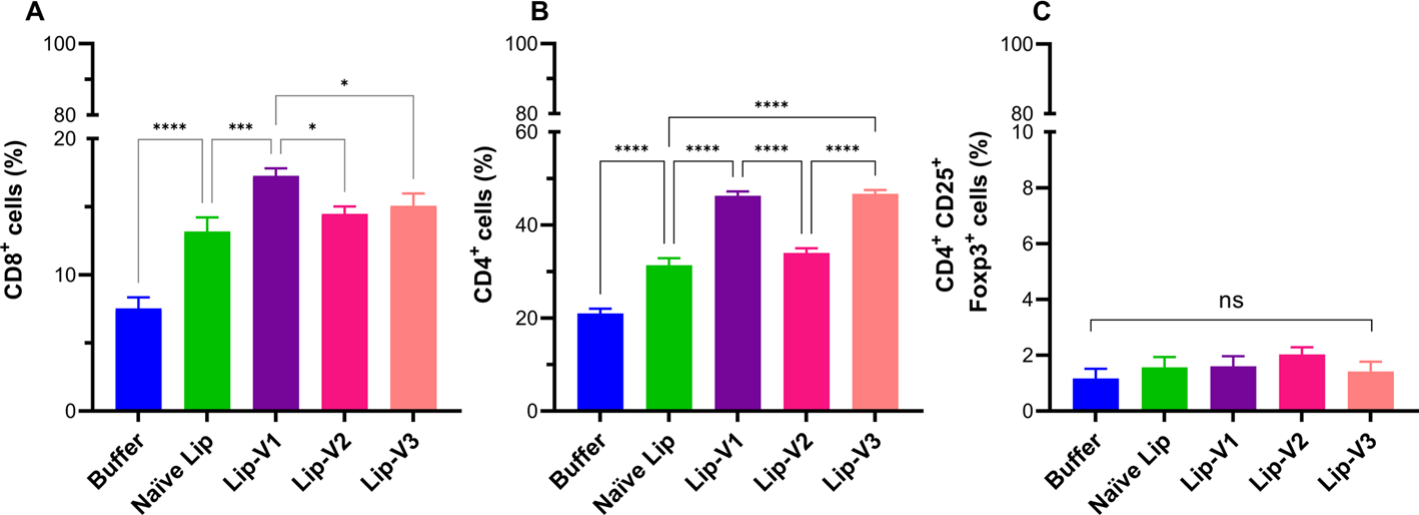
Analysis of the frequency of T cell subpopulations. Isolated CD8^+^ T cells **A**, CD4^+^ T cells **B**, and CD25^+^ FoxP3^+^ Treg cells **C** from spleen of vaccinated mice. On day 30th post final vaccination, three mice per group were sacrificed. Splenocytes were isolated and stimulated in vitro with V1, V2, and V3 peptides. Flow cytometry was used to study cells labeled with fluorescently tagged antibodies. Data are presented as mean standard deviation (*n* = 3). *Lip* Liposome; *P* > 0.05; *, *P* < 0.05; **, *P* < 0.01; *** *P* < 0.001, ****P < 0.0001

The utilization of flow cytometry was employed to analyze the proportion of T cell subgroups comprising CD4^+^ T cells, CD8^+^ T cells, and CD25^+^ FoxP3^+^ Treg cells in mice that received vaccination. As indicated in Fig. 4, animals vaccinated with Lip-V1 had higher levels of both CD4^+^ and CD8^+^ T cells than naïve liposome groups (*P* < 0.001, Fig. 4A). Furthermore, the proportion of CD4 ^+^ and CD8 ^+^ T cells in isolated cells from mice receiving Lip-V1 was greater than in Lip-V3 and control groups (*P* < 0.0001, Fig. 4B). Additionally, Lip-V2 results did not show a significant increase in the percentage of CD4^+^ and CD8^+^ T cells compared to naïve liposomes. As depicted in Fig. 4C, compared to naïve liposomes and the buffer group, the proportion of Treg cells in mice immunized with Lip-Vs remained unchanged.

### The intracellular cytokines secretion

**Fig. 5:**
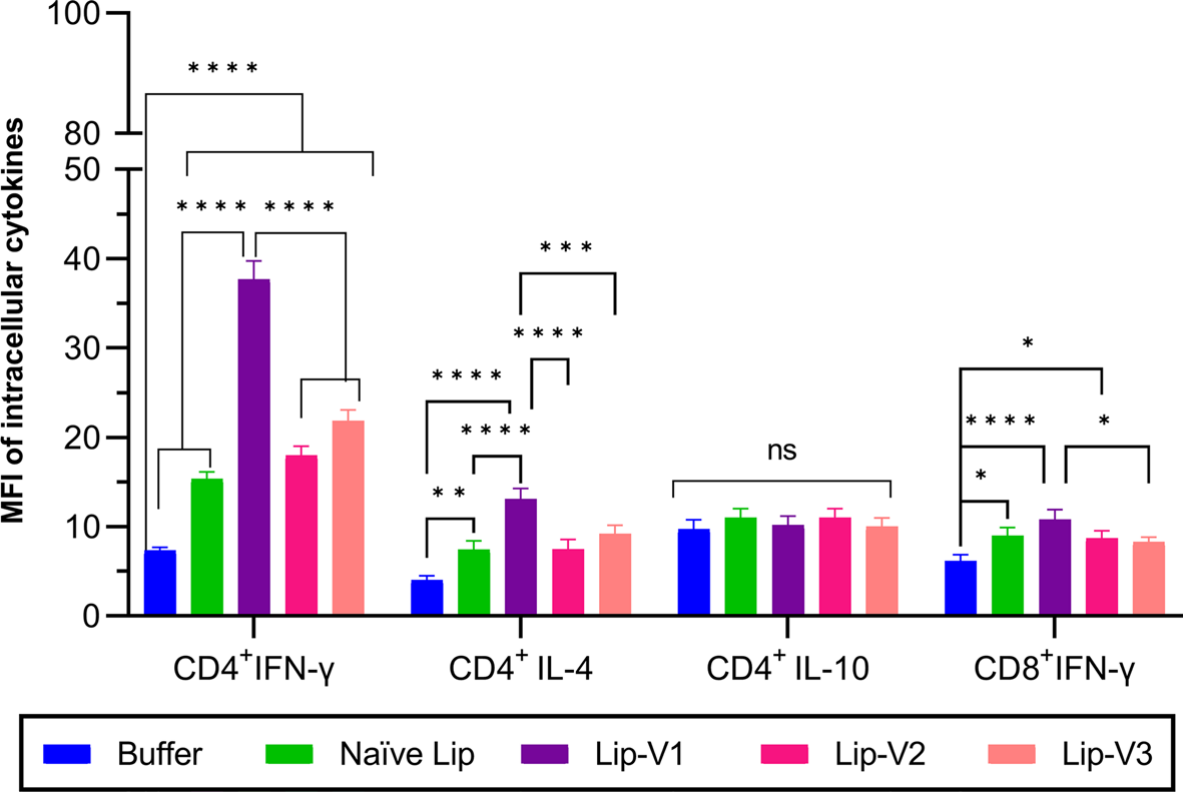
Mean fluorescence intensity (MFI) of intracellular cytokines. On the day 30th post final vaccination, three mice per group were sacrificed, and splenocytes were isolated and stimulated with V1, V2, and V3 peptides. Then the cells were stained with antibodies targeting intracellular cytokines. Flow cytometry was used to measure the MFI of various cytokines. The intracellular cytokines in CD8^+^ and CD4^+^ splenocytes were measured. This involved assessing the geometric mean fluorescence intensity of IFN-γ in CD8^+^ cells, IFN-γ in CD4^+^ cells, IL-4 in CD4^+^ cells, and IL-10 in CD4^+^ Foxp3^+^ cells of mice that had received therapeutic vaccines. Data are shown as mean ± standard deviation (*n* = 3). Lip, Liposome, *P > 0.05, **P < 0.05, ***P < 0.01, ****P < 0.001, *****P < 0.0001

The assay for intracellular cytokines showed that the CD8 ^+^ cytotoxic T lymphocytes (CTL) in the splenocytes of vaccinated mice produced significantly higher levels of IFN-γ compared to the group that received only the buffer solution (*P* < 0.0001) (Fig. 5). However, Lip-V1 triggered significantly greater levels of IFN-γ cytokine production in comparison to Lip-V3 (*P* < 0.0001). Additionally, the quantity of IFN-γ produced by CD4 ^+^ cells was notably higher in Lip-V1 and Lip-V2 groups than in the groups receiving empty liposomal (Naïve liposome) and buffer. The level of IL-4 generated by CD4 ^+^ cells was significantly greater in the Lip-V1 group compared to the other groups (*P* < 0.0001). Moreover, there was no significant increase observed in the level of IL-10 cytokines.

### Analysis of TILs in the tumor site

**Fig. 6:**
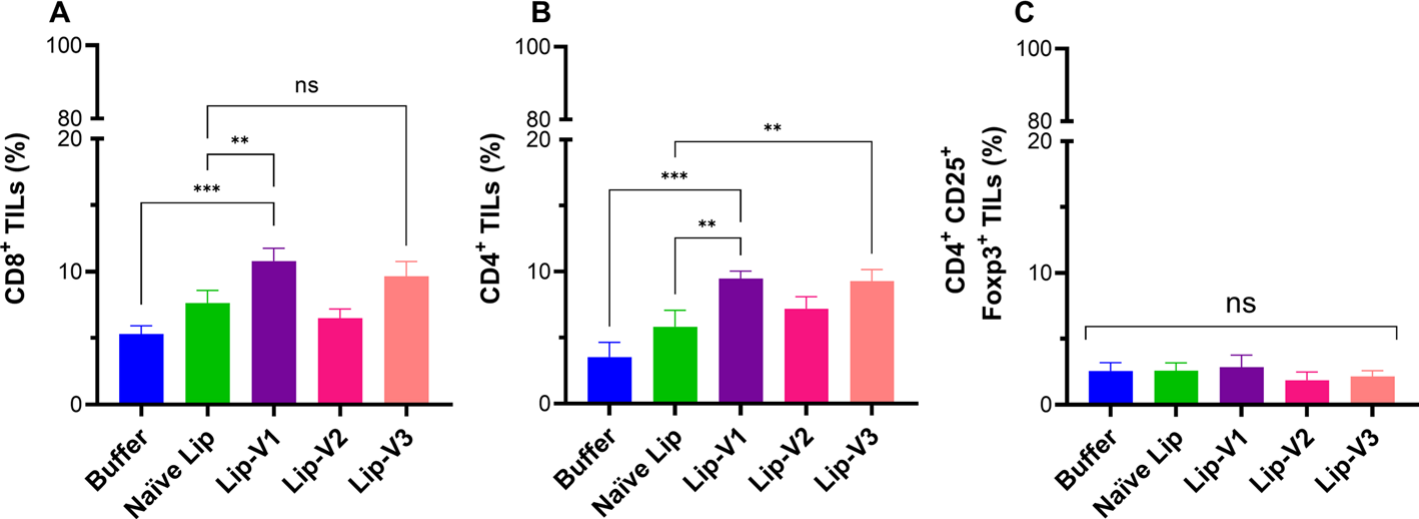
Flow cytometric evaluation of TILs in the tumor sites of C57BL/6 mice. Statistical analysis for the percentage of **A** CD3^+^ CD8^+^ TILs, **B** CD3^+^ CD4^+^ TILs, and **C** CD4^+^ CD25^+^ FoxP3^+^ TILs. The statistical analysis of the data is presented as Mean ± SD with a sample size of n = 3. The level of significance is denoted as * *P* < 0.05, **P < 0.01, ***P < 0.001, and ****P < 0.0001 indicating a significant difference between groups

In contrast to the buffer and naïve liposome group, therapeutic group injected with liposomal V1 peptide showed an increased frequency of CD8^+^ TILs in the tumor site, (Fig. 6A) (*P* < 0.001). It was also found that both liposomal V1 and V3 peptides significantly increased the level of CD4^+^ TILs (Fig. 6B) (*P* < 0.01). In addition, there was no alteration in the proportion of CD25^+^ FoxP3^+^ Treg cells in the tumor site (Fig. 6C).

### Cytokine assays

**Fig. 7:**
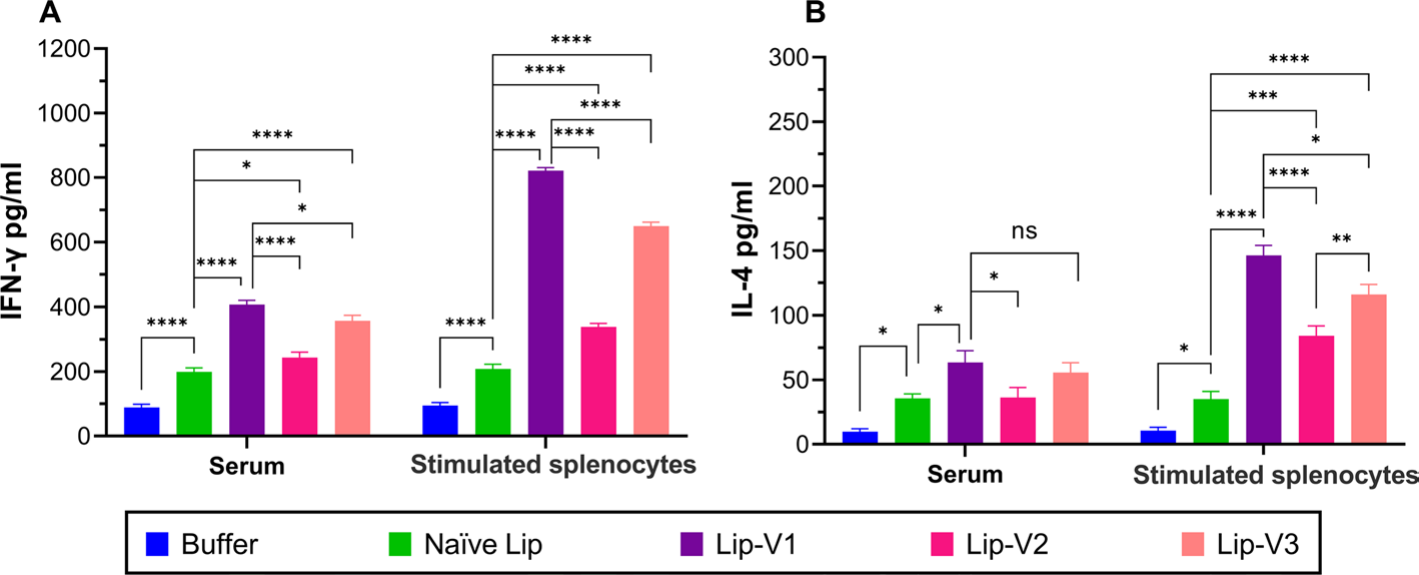
The levels of cytokines in the serum and stimulated splenocytes of vaccinated mice. The concentration of **A** IFN-γ, and **B** IL-4 concentrations were measured by ELISA The data is presented as ± standard deviation (*n* = 3). LN, lymph nodes, ns, *P > 0.05, **P < 0.05, **P < 0.01, ***P < 0.001, ****P < 0.0001, respectively, denoting the level of statistical significance

The ELISA was used to measure cytokines in the serum of mice that were vaccinated. As per Fig. 7, the levels of IFN-γ were higher in groups of animals that were vaccinated with liposomal formulations of the V1 and V3 peptide and splenocytes stimulated with V1 and V3 peptides when compared to other treatment groups (*P* < 0.0001). However, IFN-γ cytokine production was increased more by Lip-V1 than by Lip-V3 (*P* < 0.05) and other groups (*P* < 0.0001) (Fig. 7A). In contrast, Lip-V1 and Lip-V3 vaccinated mice with stimulated splenocytes showed higher levels of IL-4 than other groups (*P* < 0.0001) according to Fig. 7B. Specifically, the concentration of IL-4 in the Lip-V1 group was significantly greater than in the Lip-V3 group (*P* < 0.05). However, there was no significant difference in the level of IL-4 in mice treated with the Lip-V2 formulation (*P* > 0.05).

### Cytotoxicity assay

**Fig. 8:**
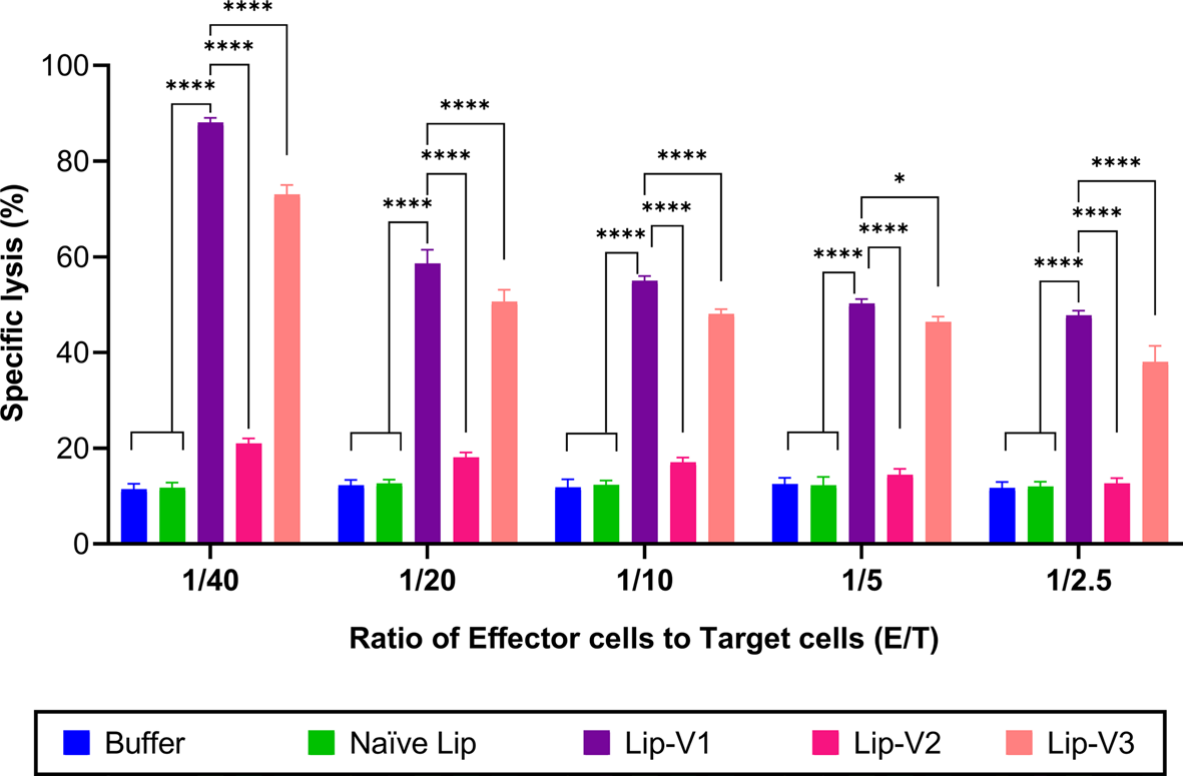
The assay for cytotoxic T lymphocytes. This assay evaluated the induction of a targeted CTL response for the removal of tumor cells using an in vitro CTL activity test. The test involved co-incubating B16F10 cells, which express VEGFR-2 (the target cells), with splenocytes in different ratios (the effector cells) and tagging them with calcein AM. The results are presented as means ± SD (*n* = 3), with E representing the effector cells and *T* the target cells. One-way ANOVA was used to analyze the data, and statistically significant differences were denoted as follows: *P < 0.05, **P < 0.01, ***P < 0.001, ****P < 0.0001

The results of in vitro splenocyte-mediated lysis demonstrated that splenocytes from vaccinated mice were more effective against B16F10 cells in an upward trend (as shown in Fig. 8). The data indicated that both V1 and V3 liposomal formulations significantly increased the specific CTL response against B16F10 cells (*P* < 0.0001). However, Lip-V1 showed better performance than Lip-V3, inducing greater CTL production and causing higher levels of specific toxicity against B16F10 cells (*P* < 0.0001). On the other hand, the Lip-V2 formulation was not capable of inducing specific toxicity against tumor cells.

### Effects of nanoliposomal formulations on antitumor immunity

**Fig. 9:**
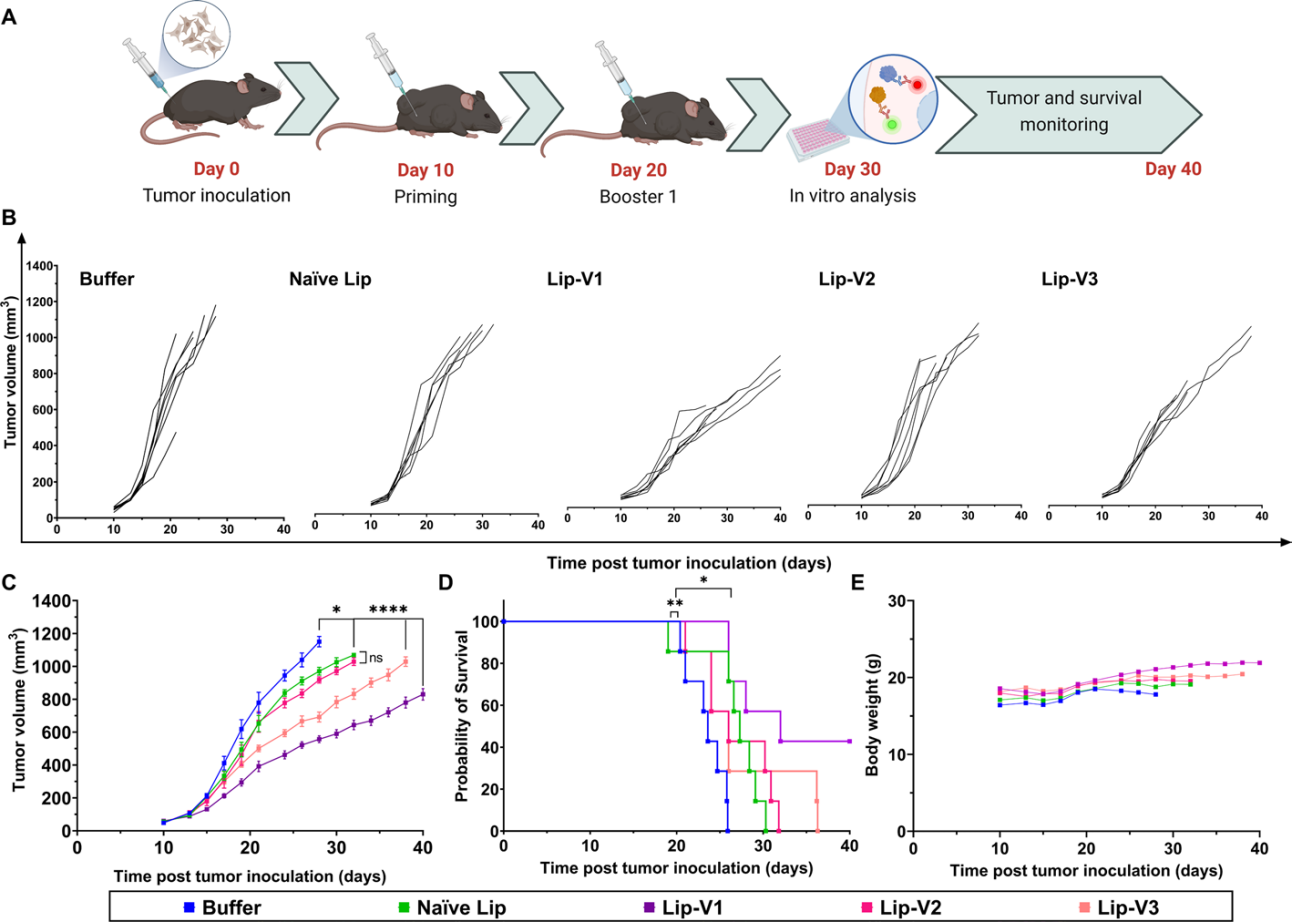
The anti-tumor efficacy of different formulations on B16F10 tumor-bearing mice. C57BL/6 mice were injected subcutaneously with B16F10 cells (5 × 105), and tumor development and survival were monitored for 40 days. Nanoliposomal peptides were found to significantly improve tumor development and survival outcomes in comparison to mice receiving buffer and naïve liposome. The experimental schedule is shown in **A**, and the tumor volume (mm^3^) of each mouse in each vaccinated and control group is shown in **B**. The average tumor growth rate in all treated groups is shown in **C**, while survival is displayed in **D**, and the average body weight of all mice is shown in **E**. The data are expressed as mean ± SEM (*n* = 7). Statistically significant differences are displayed as follows: ns, * *P* > 0.05, ** *P* < 0.05, *** *P* < 0.01, **** *P* < 0.0001

Seven tumor-bearing mice from each group were monitored for the anti-tumor effects of treatment with different formulations. The administration of liposomal V1 and V3 peptides considerably slowed the growth of the tumor and increased mouse survival rates compared to the Lip-V2 and control groups, according to the analysis of the tumor growth curve (*P* < 0.001) (Fig. 9). Additionally, the Lip-V1 formulation showed outstanding results in terms of slowing tumor development and extending survival. Naive liposomes showed improvement against tumors than the buffer formulation, as predicted (*P* < 0.05). Furthermore, the mouse survival analysis revealed that the Lip-V1 and Lip-V3 formulations increased the survival times of mice with tumors. However, as compared to the control group, Lip-V1 had the greatest survival time (*P* < 0.05) among all formulations (three mice were alive in the Lip-V1 group until the end of the study while in other groups no mice were alive). Table 3 shows the statistics for each group's increased life span (ILS), median survival time (MST), time to endpoint (TTE), and percentage of tumor growth delay (TGD).

**Table 3:** The efficacy of the treatments in the B16F10 tumor-bearing mice

Groups	TTE^a^	TGD (%)^b^	MST^c^	ILS (%)^d^
Buffer	23.5 ± 2.0	–	23.6	–
Naïve lip	26.7 ± 3.4	13.4	27.3	17.4
Lip-V1	33.1 ± 6.2	40.8	32.0	39.1
Lip-V2	26.9 ± 3.9	14.1	26.0	13.0
Lip-V3	27.4 ± 6.0	16.2	26.0	13.0

^a^Time to reach the end-point

^b^Tumor growth delay (in comparison with the buffer group)

^c^Median survival time

^d^Increase life span

## Discussion

The concept of the current study, firstly, was to design novel VEGFR-2-derived epitope peptides restricted for human HLA-A*02:01 and secondly, was to enhance the immunogenicity of the VEGFR-2 peptides in a nanoliposomal-based formulation. When compared to control groups, we discovered that vaccination with the liposomal formulation may increase the capacity of immune responses, enhance the level of IFN-γ, and increase the frequency of CD8^+^ and CD4^+^ T cells in vaccinated mice. In addition, this formulation resulted in a remarkable reduction in tumor volume and enhanced survival in a murine melanoma model.

One of the main causes of the poor clinical effectiveness of cancer immunotherapy is believed to be the loss or downregulation of HLA molecules in the tumor cells (Ryschich et al. 2005; Khong and Restifo 2002). Under these situations, one strategy to tackle such challenges is the development of vaccines against vascular endothelial cells produced in tumor tissues. Vascular endothelial cells, which express HLA molecules persistently, perform critical roles in tumor formation and progression. Furthermore, melanoma cancer cells have been shown to express VEGFR-2 and are expected to be the target of CTL (Mehnert et al. 2010). Several clinical studies have demonstrated that VEGFR peptide vaccination can be an effective way for the treatment of many cancers including pancreatic, colorectal, glioblastoma, etc. (Miyazawa et al. 2010; Hazama et al. 2014; Tamura et al. 2020; Suzuki et al. 2013; Iinuma et al. 2014).

We designed several mouse MHC class I and human HLA-A*02:01 restricted peptide epitopes of the VEGFR-2 protein using immunoinformatic tools since the HLA-A*02:01 allele is presenting at high frequencies in all ethnic populations (Song et al. 2013). Among all sequences, we selected the three most potent peptides including V1: YMISYAGMV, V2: YTVILTNPI, and V3: IQAANVSAL, according to different selection criteria that are mentioned in Table 1.

This study aimed to enhance the effectiveness of the VEGFR-2 peptide vaccine by using a liposomal formulation to deliver the epitope peptides. The research is the first of its kind to explore this approach for VEGFR-2 peptide vaccination. Results demonstrated that the liposomal formulations containing antigenic peptides were more effective in inducing immunological responses than untreated or naïve liposomal formulations. The improved immunogenicity is attributed to the ability of liposomes to transport peptides from injection sites to lymph nodes (as shown in Fig. 2) and to facilitate antigen presentation by APCs (Zamani et al. 2018). Several studies have emphasized the significance of liposomes as vaccine-delivery vehicles. Liposomes have been proposed as excellent carriers for the development of new vaccines due to their greater efficiency in antigens and adjuvants delivery to the immune system compartment (Zamani et al. 2018). Liposomal vaccine administration technologies provide a major advantage in terms of diversity and flexibility when compared to alternative adjuvant or antigen delivery techniques. Several studies have demonstrated the significance of liposome features is influenced by physicochemical factors such as particle size, lipid content, surface charge, and antigen or adjuvant placement, all of which may be defined and easily modified to obtain desired attributes. The phospholipid content of liposomes mainly influences their surface charge. This can be altered by incorporating charged phospholipids, such as the negatively charged DMPG used in this study. Our findings show that all liposomes containing DMPG have a negative charge, which improves formulation stability and prevents aggregation. Additionally, negatively charged liposomes are more effective than neutral ones as vaccine delivery vehicles. They also enhance antigenic peptide entrapment. During encapsulation, some peptides may attach to the surface of liposomes due to physical and ionic interactions between the negatively charged phospholipid headgroups of the liposomes and the cationic side groups of the peptide (Tandrup Schmidt et al. 2016). In addition, liposomes can encapsulate antigens and serve as a vaccine delivery method, as well as an adjuvant, and their efficacy, is affected by the amount of lipid layers, content, production process, and electric charge. (Arab et al. 2018). In this study, we performed encapsulation of peptides in liposomal formulations with high encapsulation efficiencies (around 70%) (Additional file 1: Fig. S1, Table 2).

Cancer vaccine requires the development of efficient cross-presentation. Nanoliposomes are excellent substitutes for enhancing cross-presentation as they increase the uptake of antigens by APCs, shield the antigens from degradation by intracellular proteases, and guarantee the sustained release of the target antigens (Kim et al. 2019; Du and Sun 2020). In addition, the use of pH-sensitive liposomes composed of DOPE phospholipid resulted in a higher escape of antigens from endosomes and cross-presentation of exogenous antigens through the cytosolic pathway, resulting in significantly enhanced cross-presentation of antigens (Belizaire and Unanue 2009; Yuba 2020). It has been reported that DOPE-containing liposomes, like those used in the current work, indicated both MHC class I and II-mediated antigen presentation, whereas liposomes without pH-sensitive constituents only activate MHC class II-mediated antigen presentation (Belizaire and Unanue 2009; Zamani et al. 2022).

According to prior studies, the liposomal formulation including peptide epitopes was highly successful in eliciting CTL responses in mice models (Arab et al. 2018; Yazdani et al. 2020a). Antigenic peptides included in a liposomal vaccination may successfully penetrate the draining lymph nodes and stimulate immune responses. Furthermore, several studies have demonstrated that liposomal peptide vaccines are more effective in inducing the anti-tumor immune response than peptide vaccines alone (Yazdani et al. 2020b). We found that both Lip-V1 and Lip-V3 formulations were successfully capable of inducing strong CD8^+^ CTL and CD4^+^ helper cell responses and considerably increased production of IFN-γ and IL-4. Additionally, these two formulations significantly decreased tumor volume and improved survival in vivo. However, the response that was induced by the Lip-V1 formulation was remarkably higher than Lip-V3. Hence, the Lip-V1 formulation could be considered a potential candidate for further research.

## Conclusion

Taken together, our research showed that Lip-V1, a nanoliposomal formulation containing the VEGFR-2 peptide, dramatically increased the amount of T cell subpopulations, decreased tumor size, and extended the survival time of tumor-bearing mice. These results imply that designing a cancer peptide vaccine should address strengthening the peptide delivery strategy to prevent its degradation, which appears to be provided by nanoliposomes. On the other hand, the immune response and anticancer properties of this nanoliposomal peptide vaccine could be improved by using adjuvants and combining them with other effective treatment strategies such as chemotherapy, that is chemoimmunotherapy.

## Methods

### Materials

Dimyristoylphosphoglycerol (DMPG), Dioleoylphosphatidylethanolamine (DOPE), and Dimyristoylphosphatidylcholine (DMPC) were purchased from Avanti Polar Lipid (Alabaster, USA). Cholesterol was purchased from Sigma-Aldrich (Steinheim, Germany). VEGFR-2 peptides, V1 (YMISYAGMV, purity > 99.15%), V2 (YTVILTNPI, purity > 97.30%), and V3 (IQAANVSAL, purity > 96.03%) were synthesized by China Peptides Co. (Shanghai, China). 1,1′-dioctadecyl-3,3,3′,3′-tetramethylindotricarbocyanine iodide (DiR) was purchased from Invitrogen (Carlsbad, CA). Flow cytometry antibodies, PMA/ionomycin cocktail, and IFN-γ and IL-4 ELISA kit (ELISA MAX^™^ Deluxe), were purchased from BioLegend (San Diego, CA). All of the solvents and reagents were molecular grade.

### Animals

Female C57BL/6 mice aged between 4 and 6 weeks were procured from Royan Institute, Tehran, Iran. The mice received ethical and humane treatment as per institutional guidelines, and all protocols were approved by the Institutional Ethical Committee and Research Advisory Committee of Mashhad University of Medical Sciences (MUMS). The procedures adhered to animal welfare guidelines and were conducted under Ethic No. IR.MUMS.MEDICAL.REC.1400.105.

### Cell lines and media

The cell lines used in this study were obtained from the Pasteur Institute Iran cell bank. B16F10 cells were grown in Dulbecco’s modified Eagle’s medium (DMEM) while NIH-3T3 cells were cultured in RPMI-1640 medium. Both cell media were supplemented with 10% fetal bovine serum and 100 U/mL penicillin and 100 μg/mL streptomycin (Gibco, UK). The cells were incubated in a 5% CO2 incubator at 37 °C.

### Designing of antigenic peptides

The VEGFR-2 antigenic peptides were designed by in silico analysis. The murine and human VEGFR-2 protein sequences were obtained from the UniProt database. After the alignment of mouse and human sequences, the common regions of the protein were used to design VEGFR-2 epitopes. Different selection criteria were used to find T-cell-binding epitopes derived from the VEGFR-2 protein, which resulted in the adoption of three peptides, each of which was 9 amino acids long. The existence of a high number of known MHC class I-restricted epitopes within a short sequence was the first criterion. The prediction of proteasome cleavage was the second criterion, and the prediction of peptide binding to the TAP transporter was the third criterion. The appropriate peptide sequences that simultaneously have the highest binding affinity to mouse MHC I (H-2-Db, H-2-Kb) and human HLA-A*02 were designed. Four different software programs were used to determine these epitopes characteristics including NetCTLpan (https://services.healthtech.dtu.dk/service.php?NetCTLpan-1.1, the Immune Epitope Database (IEDB) (https://www.iedb.org), NetMHC 4.0 (https://services.healthtech.dtu.dk/service.php?NetMHC-4.0), and PickPocket 1.1 (https://services.healthtech.dtu.dk/service.php?PickPocket-1.1) Server. Using available computer-based algorithms, three peptides from the VEGF-R2 protein were selected for synthesis and immunologic evaluation. VEGF-R2 peptides (V1: YMISYAGMV, purity > 99.15% and molecular weight (MW) of 1034.27 Da, V2: YTVILTNPI, purity > 97.30% and MW of 1033.24 Da and V3: IQAANVSAL, purity > 96.03% and MW of 886.02 Da were purchased from China Peptides Co. (Shanghai, China). Analytical high-performance liquid chromatography and mass spectrometry were used to characterize the designed peptides.

### Preparation of nanoliposomes containing peptides

Nanoliposomes containing antigenic peptides were prepared using the lipid film hydration method as previously described (Nikpoor et al. 2015) (Scheme 1). Firstly, a lipid film composed of DMPC:DMPG:DOPE:Chol at a molar ratio of 60:8:20:12, with a lipid concentration of 50 mM was prepared. For this, the appropriate amounts of phospholipids (dissolved in chloroform) was combined in sterile glass tubes. For liposomal formulations containing VEGF-R2 peptides in DMSO, 100 μg/ml of each peptide was added to the lipid mixture. The organic solvents were removed using a rotary evaporator (Heidolph, Germany) and a freeze-drier (VD-800F, Taitech, Japan). The remaining lipid film was hydrated with HEPES buffer (10 mM, pH 7.2) and 10% sucrose at 40 °C, and then thoroughly dispersed in the solution by vortexing. The resulting multilamellar vesicles (MLVs) were sonicated at 40 °C to form small unilamellar vesicles (SUVs). In order to remove unentrapped peptides, liposomes were finally centrifuged using Amicon Centrifugal Filter columns with 10kD molecular weight cut-off filters (Merck KGaA, Darmstadt, Germany). The final nanoliposomal formulations (Lip-Vs) were sterilized by filtration through a 0.22 μm microbial syringe filter and stored at 4 °C under nitrogen gas.

### Characterization of nanoliposomes

The concentration of phospholipid was determined using the Bartlett phosphate method (Bartlett 1959). The particle characterization including size (nm), zeta potential (mV), and polydispersity index (PDI) was performed by a dynamic light scattering (DLS) instrument (Nano-ZS; Malvern, Southborough, UK). Transmission electron microscopy (TEM) (Zeiss, Jena, Germany) was used to determine the morphological characteristics of liposomes. The amounts of VEGF-R2 peptides present in nanoliposomes was determined using HPLC, while the percentage of peptide encapsulation efficiency was determined using a 10kD Amicon Centrifugal Filter column. To do this, a sample of both the post-Amicon filtrate and nanoliposomes were dissolved in OG (Octyl glucoside 200 μg/ml) and injected into an HPLC machine (KNAUER, Germany) that utilized a C18 column (Nucleosil, 150 × 4.6 mm) with an H_2_O:0.1% trifluoroacetic acid mobile phase (99.9:0.1 (*v*/*v*), eluent A). The eluent gradient was set to 80% eluent A over 3 min. The peptides were identified by measuring the absorbance at 220 nm at a flow rate of 1 mL/min. The HPLC method was used to calculate the percentage of encapsulation efficiency (% EE) of peptides, and it was determined using the following formula:

$$\mathrm{\%\, Encapsulation}=\frac{Total \,amount \,of \,peptide - Amount\, of\, peptide\, in \,filtrate }{Total\, amount \,of\, peptide} \times 100$$

### In vivo imaging assay

In order to analyze the biodistribution and accumulation of nanoliposomes in lymph nodes, fluorescently labeled liposomes were prepared as described elsewhere (Mirzavi et al. 2022). For this, DiR fluorescent dye with a molar ratio of 0.2% was used in the preparation of the labeled liposomal formulations (DMPC:DMPG:DOPE:Chol at a molar ratio of 60:8:20:12). The prepared liposome formulation, was subcutaneously injected into mice. At several time points (0, 24, 48, 72, and 96 h after injection), the images were taken (Kodak in vivo imaging system F pro, Rochester, USA). At the end of the experiment, the mice were euthanized, and their lymph nodes, spleen, liver, hands, and feet were examined using ex vivo imaging. The fluorescent DiR dye was used with excitation at 690 nm and emission at 780 nm.

### Isolation of PBMCs and in vitro cellular uptake assay

The cellular uptake of nanoliposomes was assessed using flow cytometry with peripheral blood mononuclear cells (PBMCs) according to the method outlined in our previous research studies (Dehghan-Manshadi et al. 2021; Zamani et al. 2019). Approximately 1 mL of fresh whole blood was collected from the mice’s hearts into heparinized tubes, diluted with an equal volume of phosphate-buffered saline (PBS), and used to isolate PBMCs using Ficoll/Hypaque (Sigma, St. Louis, Missouri, USA) in accordance with the manufacturer’s instructions. The isolated cells were then centrifuged at 800 g for 20 min at room temperature. PBMCs were collected from the interface, mixed with PBS, and then centrifuged twice. After that, 100 μl of DiR-labeled liposomes were added directly to the cells and incubated for 2 h at either 4 or 37 °C. Untreated PBMCs were also used as a control. The cells were then washed twice with PBS to remove any remaining free liposomes. Finally, the cells were suspended in 300 μl PBS and analyzed using a flow cytometer (BD FACSCaliburTM, BD Biosciences, San Jose, USA).

### Tumor inoculation and immunization of C57BL/6 mice

Xenograft tumor model created by subcutaneous inoculation of B16F10 cells (5 × 10^5^) in Female C57BL/6 mice (4–6 weeks). On day 10 post-tumor inoculation, the tumors were detectable and had grown to a size of 3 mm. Then, tumor-bearing mice were randomly assigned to 5 treatment groups (*n* = 10 mice per group) as follows: (i) buffer, (ii) naïve liposome, (iii) liposomal V1 (Lip-V1), (iv) Lip-V2, and (v) Lip-V3. Mice were subcutaneously vaccinated twice, at 10 days intervals, using various liposomal formulations (Scheme 1). Each liposomal formulation with a lipid dose of 5 μmol per mouse was administered. Naïve liposome and HEPES-sucrose 10% buffer, were given to control groups. On the 30th day after tumor inoculation, three mice from each group received a booster and were used for in vitro experiments. The remaining mice in each group were monitored for tumor size, weight loss, and survival time in vivo.

### Splenocytes isolation

Following 10 days of the final vaccination, three mice from each group were euthanized by injecting 100 μL of ketamine-xylazine solution (100 mg/kg ketamine and 10 mg/kg xylazine) (Xu et al. 2007). The spleens were collected and gently homogenized with a sterile 3 cc syringe (2 cc) and filtered through a cell strainer under sterile conditions. The erythrocytes were eliminated using ACK buffer (0.15 M NH_4_Cl, 1.0 M KHCO_3_, and 0.1 mM Na_2_EDTA). The viable splenocytes were counted using trypan blue (0.4% *w*/*v*) (Gibco) and suspended in 10% FBS-supplemented RPMI-1640 media.

### The frequency of T cell sub-populations and intracellular cytokines assays by flow cytometry

To conduct the intracellular cytokine assay, the splenocytes were isolated and cultured in RPMI-1640 medium with 10% FBS at a concentration of 10^6^ cells/mL. The cells were then stimulated with 10 μg/mL of each VEGFR-2 peptide at 37 °C for 12 h. After incubation, 1 µl per milliliter of brefeldin A solution (from BioLegend, San Diego, CA) was added to the cell medium and incubated for another 4 h. Flow cytometry was used to analyze the results. Splenocytes (10^5^ cells/mL) were stimulated for 4 h at 37 °C using a mixture of 1 μL/mL PMA and ionomycin as a positive control. All cells were washed with a staining solution (2% FBS in PBS) and stained using flow cytometry antibodies (BioLegend, San Diego, USA), as explained previously (Zamani et al. 2022). Splenocytes (10^5^ cells) were briefly stained for 30 min at 4 °C in separate tubes with surface antibodies (anti-CD4-PE-cy5 or anti-CD8a-PE-cy). The cells were then fixed with Cytofix/Cytoperm solution and washed with staining buffer (PBS containing 2% FCS). After being washed twice with Perm/Wash TM solution, fixed cells were stained for 30 min at 4 °C with intracellular antibodies (anti-IL-10-APC, anti-IFN-γ-FITC, anti-IL-4-PE, anti-Foxp3-PE). A flow cytometer (BD FACSCaliburTM, BD Biosciences, San Jose, USA) was used to analyze the cells after washing them with Perm/Wash TM solution and suspending them in 300 μL of flow cytometry staining buffer.

### Enzyme-linked Immunosorbent (ELISA) assay

The ELISA technique was used to measure the levels of IFN-γ and IL-4 cytokines in both the blood sample and stimulated splenocytes. Briefly, blood samples were taken on the 30th day following the final vaccination, and sera were isolated using centrifugation at 4 °C for 15 min at 2000 g and were utilized to measure systemic cytokine levels. In addition, the cytokines levels secreted in response to peptide stimulation were evaluated in supernatants of cultured splenocytes (Shahbaz et al. 2020). Cells were cultured in triplicate on 24-well plates (Nunc, Denmark) at a density of 2 × 10^6^ cells/ml and stimulated with 10 μg/ml of VEGFR-2 peptides. Additionally, cells were stimulated with 2% *V*/*V* of PHA as positive or medium as negative controls, respectively. All cells were at 37 °C for 72 h. After incubation time supernatants were collected. Evaluation of cytokine levels was carried out using the supernatants according to the manufacturer’s protocol of ELISA MAX^™^ Deluxe Set Mouse IL-4 and IFN-γ kit, (BioLegend. San Diego, CA).

### In vitro* cytotoxicity assay*

In vitro splenocyte-mediated lysis was evaluated by Calcein-AM staining. Briefly, on the 30th day of the study, three mice per group were sacrificed, and splenocytes were collected as effector cells. The B16F10 cells and NIH3T3 cells (used as positive and negative target cells for expression of VEGFR-2) were treated with 12.5 M calcein acetoxymethyl (Calcein-AM, Invitrogen, USA), for 1 h at 37 °C in the dark. Following incubation, targeted cells (1.2 × 10^5^ cell/well) were co-cultured with a cellular dilution of effector cells (splenocytes) at concentrations ranging from 8 × 10^5^ to 5 × 10^4^ cells/ml (various effector cell to tumor cell ratios include: 1/40, 1/20, 1/10, 1/5, and 1/2.5). For maximum and minimum lysis, Triton X-100 (2%) and culture media were used, respectively, and incubated for 4 h at 37 °C. A fluorescent plate reader was used to measure the intensity of the fluorescence at 485 nm excitation and 538 nm emission (PerkinElmer Instruments Inc., Massachusetts, USA). The average value of the specific lysis of triplicate wells was calculated using the subsequent equation:

$$\%\, Specific \,lysis=\frac{\mathrm{release\, by \,CTLs }-\mathrm{ minimum\, release}\mathrm{by \,targets}}{\mathrm{maximum\, release\, by \,targets }-\mathrm{minimum\, release\, by \,targets}} \times 100$$

### Analysis of tumor-infiltrated lymphocytes (TILs)

A flow cytometer assay was used to analyze intracellular cytokine production and lymphocyte infiltration of the tumor. Three mice from each group were sacrificed 10 days following the final inoculation. immediately, tumors were taken out and washed with sterile PBS. The tissue was manually minced with a scalpel and then subjected to 1 h enzymatic digestion at 37 °C with gentle mixing utilizing 2 mg/mL collagenase type I solution (Gibco) mixed with 10 mM PBS (pH 7.4). Following incubation, the digest was quenched by the addition of 10% FBS-supplemented RPMI-1640 media. After filtering the digested tissue via a cell strainer, it was centrifuged at 1500 RPM for 10 min. Trypan blue staining (0.4%, *w*/*v*) was used to quantify the total number of tumor cells after the pellet was resuspended in flow cytometry staining solution. Surface and intracellular markers of T cells were examined by flow cytometry in the TILs.

### In vivo* anti-tumor monitoring*

The therapeutic effect of the treatment strategy was evaluated in the B16F10 xenograft melanoma model. Seven mice in each group were followed up to 40 days post-tumor inoculation. The size of the tumor and the mice’s weight were frequently measured. The following formula was used to determine the tumor volume: (length × width × height) × 0.52) (Huang et al. 2009). The mice were sacrificed if the weight loss was < 15% of their initial weight or the size of tumors was ≥ 1000 mm^3^.

### Statistical analysis

Two-way analysis of variance (ANOVA) and Tukey's post-test were used for statistical analysis. To compare survival curves, log-rank (Mantel-cox) tests were used on the survival data. *P* < 0.05 was regarded as significant. All data were analyzed using Graph Pad Prism 6 software.

## Supplementary Information

**Additional file 1: Figure S1.** Determination of V1, V2, and V3 peptides encapsulation efficiencies by HPLC: (A. Blue) Standard free V1 peptide eluted with a retention time of 1.033 minutes, (A. Purple) The extent of free V1 peptide in the post-Amicon filtrate, (A. Green) The V1 peptide spiked to empty liposomal formulation, (A. Black) The extent of encapsulated V1 peptide in liposomal formulation (Lip-V1), (B. Blue) Standard free V2 peptide eluted with a retention time of 1.083 minutes, (B. Purple) The extent of free V2 peptide in the post-Amicon filtrate, (B. Green) The V2 peptide spiked to empty liposomal formulation, (b. Black) The extent of encapsulated V2 peptide in liposomal formulation (Lip-V2), (C. Blue) Standard free V3 peptide eluted with a retention time of 1.083 minutes, (C. Purple) The extent of free V3 peptide in the post-Amicon filtrate, (C. Green) The V3 peptide spiked to empty liposomal formulation, (C. Black) The extent of encapsulated V3 peptide in liposomal formulation (Lip-V3).

## Data Availability

Data will be made available on request.
