# Supplementary material for: Nanoliposomal VEGF-R2 peptide vaccine acts as an effective therapeutic vaccine in a murine B16F10 model of melanoma
Source: Cancer Nanotechnol. 2023 Jun 14;14(1):62. doi: 10.1186/s12645-023-00213-7 (PMC10264216; doi:10.1186/s12645-023-00213-7)
Supplement: Supplementary file 1 — Additional file 1: Figure S1. Determination of V1, V2, and V3 peptides encapsulation efficiencies by HPLC: (A. Blue) Standard free V1 peptide eluted with a retention time of 1.033 minutes, (A. Purple) The extent of free V1 peptide in the post-Amicon filtrate, (A. Green) The V1 peptide spiked to empty liposomal formulation, (A. Black) The extent of encapsulated V1 peptide in liposomal formulation (Lip-V1), (B. Blue) Standard free V2 peptide eluted with a retention time of 1.083 minutes, (B. Purple) The extent of free V2 peptide in the post-Amicon filtrate, (B. Green) The V2 peptide spiked to empty liposomal formulation, (b. Black) The extent of encapsulated V2 peptide in liposomal formulation (Lip-V2), (C. Blue) Standard free V3 peptide eluted with a retention time of 1.083 minutes, (C. Purple) The extent of free V3 peptide in the post-Amicon filtrate, (C. Green) The V3 peptide spiked to empty liposomal formulation, (C. Black) The extent of encapsulated V3 peptide in liposomal formulation (Lip-V3). [file 12645_2023_213_MOESM1_ESM.docx]

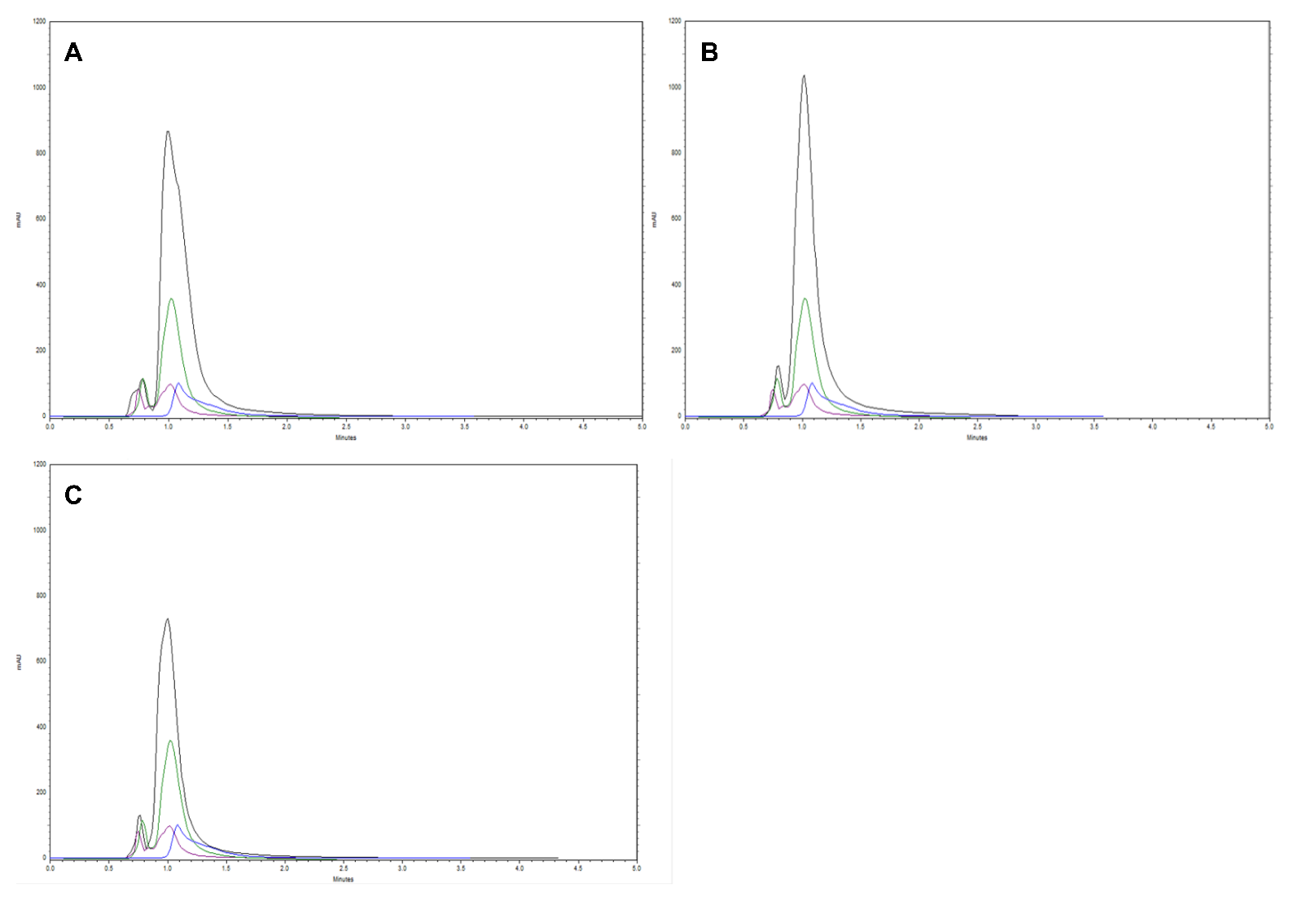


**Figure S1**. Determination of V1, V2, and V3 peptides encapsulation efficiencies by HPLC: (A. Blue) Standard free V1 peptide eluted with a retention time of 1.033 minutes, (A. Purple) The extent of free V1 peptide in post-Amicon filtrate, (A. Green) The V1 peptide spiked to empty liposomal formulation, (A. Black) The extent of encapsulated V1 peptide in liposomal formulation (Lip-V1), (B. Blue) Standard free V2 peptide eluted with a retention time of 1.083 minutes, (B. Purple) The extent of free V2 peptide in post-Amicon filtrate, (B. Green) The V2 peptide spiked to empty liposomal formulation, (b. Black) The extent of encapsulated V2 peptide in liposomal formulation (Lip-V2), (C. Blue) Standard free V3 peptide eluted with a retention time of 1.083 minutes, (C. Purple) The extent of free V3 peptide in post-Amicon filtrate, (C. Green) The V3 peptide spiked to empty liposomal formulation, (C. Black) The extent of encapsulated V3 peptide in liposomal formulation (Lip-V3).
